# Is Childhood Socioeconomic Status Independently Associated with Adult BMI after Accounting for Adult and Neighborhood Socioeconomic Status?

**DOI:** 10.1371/journal.pone.0168481

**Published:** 2017-01-17

**Authors:** Gregory Pavela

**Affiliations:** School of Public Health, University of Alabama, Birmingham, Alabama, United States of America; TNO, NETHERLANDS

## Abstract

Childhood socioeconomic status (SES) is inversely associated with adult weight in high income countries. Whether the influence of childhood SES on adult weight is best described using a critical period model or an accumulation of risk model is not yet settled. This research tests whether childhood SES is associated with adult BMI and likelihood of obesity independent of adult socioeconomic status and neighborhood characteristics. Data on individual childhood and adult characteristics come from the Health and Retirement Study (N = 13,545). Data on neighborhood characteristics come from the 2000 Decennial Census and American Community Survey (2005–2009). In the fully adjusted models, perceived financial hardship before the age of sixteen and having a father who was unemployed are associated with higher BMI among males and, among females, paternal education remains associated with adult BMI. However, childhood SES is not associated with likelihood of obesity after fully adjusting for adult SES and neighborhood characteristics, suggesting that the direct effects of early childhood SES on BMI are small relative to the other factors associated with obesity in adulthood.

## Introduction

Childhood socioeconomic status (SES) is a probable early marker for adult obesity in high-income countries [1]. Whether childhood SES influences adult weight directly through mechanisms such as biological imprinting, or indirectly via an accumulation of risk exposures, is not yet settled. A better understanding of the relationship between childhood SES and adult weight provides insight into how and when SES is embodied as obesity in the life course [2]. This paper draws upon the empirical and theoretical work of the life-course and ecological perspectives on adult health and tests whether childhood SES is associated with adult body mass index (BMI) and obesity after adjusting for adult socioeconomic status and adult neighborhood characteristics.

To date, no research has explicitly tested whether childhood SES remains associated with adult BMI after adjusting for both adult socioeconomic status and neighborhood conditions. Results provide evidence that some indicators of childhood SES are associated with adult BMI independent of adult socioeconomic status.

### Obesity and Socioeconomic Status Across the Life Course

Between 2011 and 2012, 68.5% of the U.S. adult population was either overweight or obese, with increasing prevalence at older ages [3]. Given that the sample used in analyses is intended to be nationally representative of US adults aged 51 and over, it’s important to note that prevalence of obesity generally increases with age, up to about 60 years of age, at which point the prevalence of overweight or obesity declines. Specifically, among all racial/ethnic groups in the U.S. between 2011–2012, prevalence of overweight or obesity among females reached its zenith among those aged 40–59 years, with an estimated prevalence of 71.7%. Among males, the estimated prevalence is also highest among those aged 40–59 years, with a prevalence of 79.1%. Patterns of overweight and obesity at older ages are similar when disaggregated by race/ethnicity or examining the prevalence of obesity specifically (and not in conjunction with overweight) [3]. The increasing prevalence of obesity among older adults in the US is concerning given the health outcomes associated with obesity [4–7]. Indeed, the American Medical Association recently voted to classify obesity as a medical disease state, partly with the hope that such a classification would improve public health outcomes. The rapid increase in the number of overweight or obese individuals in the United States and the associated health consequences have spurred significant research to identify risk factors for obesity, ranging from genetic to cultural factors [8].

One important risk factor for obesity is SES, though the strength of the association varies across place, time and populations. Among developed countries, SES is inversely associated with weight status among children and adolescents, with parent’s education yielding the most consistent association [9]. The association is particularly strong among white females and in high GDP countries [10], though the inverse association between SES and obesity increasingly appears in nations at a lower level of per capita income [11]. The association between SES and obesity in developed countries is likely due to many factors, including variation in material resources, social-psychological resources, and reverse causation. Indicators of adult SES intended to measure variation in both access to material and social-psychological resources are thus included in the analysis, namely years of education, household income, and household wealth.

Of significant research interest is the relationship between early life SES and risk of obesity as an adult. Indeed, the examination of health trajectories across the lifecourse more generally is one of the fastest growing areas in life-course research [12]. Prior to 1998, thirty papers examined the link between childhood SES and adult weight [13]; between 1998 and 2008 there were forty-eight papers on the topic [14]. Most of this research finds childhood disadvantage (i.e., low childhood SES) to be associated with increased weight among adults [13], and more recent research tends to find a more consistent association [14]. Associations between childhood disadvantage and adult weight suggest a “long arm” of early lifecourse experiences and their influence on later weight [15, 16]. In a large review of studies that examined early markers of adult obesity, paternal occupation was one of four factors found to be “probable” early markers of adult obesity (along with maternal body mass index, childhood growth patterns, and childhood obesity). Remarkably, paternal occupation had a more consistent association with adult BMI than paternal BMI [1]. Indicators of childhood SES may be associated with adult weight through a number of mechanisms, including parental modeling of daily weight-related behaviors [17] (e.g., the consumption of energy dense foods and sedentary lifestyles), and, as discussed below, the effect of early-life SES on adult SES and the health behaviors associated with one’s achieved SES[14]. Thus in addition to paternal occupation, the present analysis includes several other measures of childhood socioeconomic status, as well as childhood health, that may be associated with future weight as an adult, including parental education [18] and financial hardship.

Life course epidemiology (LCE) has emerged as a theoretical framework to study the effects of early life events on health throughout the life course [19]. Supplemented with an evolutionary perspective, LCE recognizes that an individual’s weight is not simply the summed effect of concurrent influences on behavior; rather, the weight of any given individual is the result of evolutionary (e.g., intergenerational transmission of characteristics) and individual developmental histories (e.g., non-inherited effects as a result of environmental exposures across the life course) [20]. Within LCE, the critical period and the accumulation of risk models describe two non-mutually exclusive processes that connect early life experiences with later health within an individual’s developmental history.

The critical period model describes early life exposures that result in biological changes which increase susceptibility to disease or health problems later in life. These exposures can occur across the life course, though much focus has been on very early life exposures. For example, fetal exposure to a nutrient deficient environment can lead to the development of a “thrifty phenotype”, which may or may not subsequently match conditions of the post-natal environment [21]. When nutrient deficiency is followed by a nutrient sufficient environment, individuals with a thrifty phenotype may have an increased risk of cardiovascular disease later in life [22]. Osmond and Barker [23] review the evidence and potential mechanisms of a thrifty phenotype for several health outcomes, including hypertension and type 2 diabetes. Beyond nutritional deficiency, other early-life conditions associated with later weight-related outcomes include an energy-rich intra-uterine environment, leptin deficiency, and birthweight [24–27].

Data permitting, critical periods of exposure can be identified with great precision. Analyses of the effects of famine exposure *in utero* indicate that the effects of exposure differ markedly by trimester—fetal exposure in the first trimester is associated with an increased likelihood of obesity while exposure in the third trimester is associated with a reduced likelihood of obesity. [28]. While the present paper uses an indicator of childhood SES that cannot precisely identify a specific critical period of exposure (e.g., the effects of poor childhood SES on *in utero* exposure to energy vs. adolescent exposures to peer norms), analyses can still provide evidence that a critical period of development exists prior to age of sixteen, during which exposure to a low SES environment influences weight as an adult. Indeed, Ben-Shlomo and Kuh (19) include poor childhood SES as predictor of poor growth *in utero* and infant respiratory infections, both potential critical periods of development affecting adult lung function and disease.

In contrast to the critical period model, the accumulation of risk model hypothesizes that individuals accumulate biological experiences, sometimes referred to as “health capital”, across the life course [19]. When the accumulation of biological experiences results from correlated events, such as coming from a family with low SES and limited access to quality schools, followed by a limited access to jobs requiring a higher education, the accumulation of risk model is also known as a pathway or “chains-of-risk” model [29]. Support for the cumulative model has been found for numerous and diverse outcomes, including cardiovascular disease [30] mortality [31], hypertension [32] drug dependence [33] and cortisol dysregulation [34].

Both the critical period and accumulation of risk models describe distinct processes linking early life events with later health outcomes, but should be considered complements for policy purposes [35]. The accumulation of risk model is more amenable to post-exposure intervention efforts given the emphasis on biological imprinting in the critical period model; however, pre-exposure health efforts such as prenatal nutrition interventions can also be effective [36]. From a policy perspective, it is therefore essential to identify points along the life course that present opportunities to intervene with maximum effect. This paper extends previous research by including previously omitted but important measures of adult social conditions, neighborhood characteristics, in analyses that test whether childhood SES and health are independently associated with adult BMI and obesity.

Research that integrates lifecourse and ecological perspectives on health is rare, although there are recent examples using data from Chinese and European populations [37–40]. While the focus of the present analysis is on the potential long-term effects of childhood socioeconomic status on adult weight, previous research using an integrated life course and ecological perspective has used broader indicators of childhood well-being that include psychosocial and financial well-being (i.e., childhood adversity). Using data from the Finnish Public Sector Study, Halonen, Stenholm (39), found that childhood adversity followed by residing in a low SES neighborhood as an adult (indicated by unemployment and education levels) was associated with a more than 2-fold increase in cardiovascular risk factors; however, exposure to only one risk factor (i.e., childhood adversity or low neighborhood SES) was not associated with an increased risk of cardiovascular disease, suggesting it is the combined effect of early and later life adversity that matters for adult cardiovascular disease. The results of Slopen, Non (40) also indicate the importance of simultaneous consideration of the effects of childhood adversity and adult neighborhood SES. In their analysis of the Chicago Community Adult Health Study, childhood adversity was associated with an increase in cumulative biological risk for chronic disease (an index of eight items including waist circumference, blood pressure, and cholesterol), but only among individuals who resided in low SES neighborhoods as an adult. Finally, the results of Wen and Gu (2011) indicate that childhood conditions had particularly strong associations with cognitive functioning and mortality, even after controls for neighborhood SES. Taken together, these findings indicate the value of simultaneously considering lifecourse processes and neighborhood characteristics.

### Neighborhood Characteristics and Obesity

Socio-ecological theory posits that one’s environment, the interconnections between one’s environment, and the historical context of environmental exposures are important for individual development [41]. Neighborhood characteristics are an important part of one’s immediate environment (also known as the microsystem) and are associated with adult weight, an association that, as with individual measures of SES, is usually stronger among females than males [42–44]. The consequences of neighborhood context for health have been of interest to researchers since the 1920s, as growing urbanization increased the visibility of poor urban living conditions [45]. More recently, the deinstitutionalization of inner-city neighborhoods—the process by which job opportunities decline, working families move to better neighborhoods, and schools, housing, and community organizations deteriorate—has been of concern to sociologists [46, 47]. The de-institutionalization of neighborhoods has not only influenced the opportunity structures within city neighborhoods, but has also resulted in neighborhoods stratified by social characteristics including socioeconomic status, family structure, and, importantly, race [45].

As the importance of neighborhood characteristics have been increasingly recognized for their role in individual health as well as racial/ethnic differences in health [48], a variety of mechanisms linking neighborhood conditions to health have been proposed, including land use patterns, psychosocial context, positive peer interactions, and material resources available [43, 45, 49, 50]. Americans living in disadvantaged communities may have thus increasingly found themselves separated from the healthful aspects of advantaged communities and isolated within an obesogenic environment. These environments may contribute to obesity via a lack of material resources such as healthy food options and incentives for physical activity, or a psychosocial context that increases the risk of obesity through mechanisms related to chronic stress [43]. Given the association between neighborhood characteristics and obesity, and the general pattern of stronger associations between measures of SES and obesity among females [10], the characteristics of one’s neighborhood may play an especially important role among females in a “chains-of-risk” model linking childhood conditions to later adult weight.

However, associations between neighborhood characteristics and health, including weight, may also be due to selectivity, such that higher weight individuals are selected into lower SES neighborhoods, confounding causal estimates of the effects of neighborhood characteristics on weight or some other outcome of interest [51, 52]. Indeed, recent research has demonstrated that health can affect subsequent neighborhood context. Among a sample of Hurricane Katrina survivors, individuals who reported better health prior to Hurricane Katrina were less likely to be exposed to neighborhood deprivation in the years following Hurricane Katrina [53]. Nonetheless, evidence of a causal effect of neighborhood conditions on health and weight comes from the Moving to Opportunity Program (MTO), a randomized housing mobility experiment involving 4,600 low income families (Katz, Kling and Liebman 2000). Families who volunteered and were selected to move to lower poverty areas reported an improvement in health status, greater feelings of peace, and women were less likely to have extreme obesity and diabetes [54]. Given the evidence of both health selection [53] and causal effects of neighborhood characteristics [49], it is therefore plausible, even likely, that the association between neighborhood characteristics and adult weight has a bi-directional causal flow, and interpretation of results must include this possibility given the observational nature of the data in the analysis.

In summary, given the observed association between neighborhood characteristics and adult obesity, it is hypothesized that childhood SES will not be associated with adult BMI after adjusting for measures of adult neighborhood socioeconomic status, the built environment, and neighborhood demographics. If childhood SES is not independently associated with adult BMI, it suggests an accumulation of risk model is more consistent with the evidence linking childhood SES to adult weight than the critical period model.

## Data and Methods

To test whether childhood SES is independently associated with adult BMI after adjusting for neighborhood characteristics, I conduct an analysis of cross-sectional data predicting adult BMI from childhood conditions, neighborhood characteristics, and other individual covariates. Data for the analyses come from multiple datasets. First, data on individual characteristics come from the Health and Retirement Study (HRS), a nationally representative panel study that collects information on the economic, health, marital, and family status of the US population over the age of 50 and their spouses who were not institutionalized at their first interview and sponsored by the National Institute of Aging (grant number NIA U01AG009740) [55]. Launched in 1992, the HRS has collected nine waves of data on a biennial schedule. At the 1992 baseline, the HRS sample size was 12,652 respondents (7,704 households) with a response rate of 81.7%. Individuals included in the analysis come from the AHEAD (The Study of Assets and Health Dynamics Among the Oldest Old) cohort, born prior to 1924, the Children of Depression cohort, born 1924–1930, the HRS cohort, born between 1931 and 1941, and the War Baby cohort, born 1942–1947. All four birth cohorts were first asked about childhood conditions in 1998. Additional individual level data on adult demographics and socioeconomic status for the years 2006 and 2008 come from the Research ANd Development Corporation (RAND) HRS dataset, a cleaned and processed version of the raw data with consistent naming of variables across waves and imputations for key variables often missing in the raw data, and funded by the National Institute on Aging and the Social Security Administration [56]. The analytic sample includes individuals across a wide range of ages, and the impact on the health-and well-being may vary across such a wide age group. Nonetheless, with a majority of adults over the age of 66, the adverse health consequences of obesity for older adults makes understanding the relative contribution of early and later life experiences on BMI and obesity important.

Neighborhood characteristics come from the American Community Survey (ACS) 2005–2009 and the 2000 Decennial Census. ACS data used in the analysis describe the average characteristics of the population and housing of US census tracts over the five-year period between 2005 and 2009 [57]. All neighborhood measures come from the ACS unless other noted. Individual and neighborhood data were merged using the census tract identifier in the restricted geographic data module of the Health and Retirement Study dataset, thus neighborhood characteristics of HRS respondents represent characteristics of the census tract in which they reside. This research was approved by the University of Alabama at Birmingham Institutional Review Boards for Human Use with a protocol number of X140409004.

### Measures

#### Dependent variable-individual body mass index and obesity

Adult weight is modelled using 1) a continuous measure of individual BMI computed from technician measured weight and height in waves 8 and 9 of the HRS (years 2006–2008) and 2), as a sensitivity check, a 0/1 binary variable indicating obesity (BMI ≥ 30.). In 2006, half of the sample was randomly selected to receive an enhanced face-to-face interview, which included direct measurements of height and weight. In 2008, the other half of the sample received the enhanced face-to-face interview. The use of technician measured weight and height is a significant strength of this research. Most previous research on neighborhood characteristics and weight using nationally representative data has relied on self-reported height and weight (e.g., [42, 43].

#### Independent variables-childhood measures

Measures of childhood circumstances come from the 1998 HRS data. Parental education was measured by asking respondents “what is the highest grade of school your mother (father) completed?” Responses in years were then recoded into a series of dummy variables with categories defined as having more than a high school education (reference category), high school degree or General Educational Development (GED), less than a high school education, or missing information. Father’s occupation/family status was coded as a series of dummy variables and defined as having a father who was consistently employed (reference category), ever unemployed, absent, or missing information. Paternal job loss was measured by asking respondents if, before the age of 16, there was a time of several months or more when your father had no job. Childhood health was measured by asking respondents to consider their health from birth to age 16 and rate their health as excellent, very good, good, fair, or poor and dichotomously coded (1 = poor or fair childhood health versus good, very good, or excellent health). Childhood family SES was measured by asking respondents to rate whether their family was “pretty well off financially, about average, or poor” and dichotomously coded (1 = poor; 0 otherwise). In reporting results, this variable is referred to as financial hardship. Lastly, respondents were asked whether “financial difficulties ever cause you or our family to move to a different place” (1 = yes; 0 otherwise).

#### Independent variables-adult demographic and SES

Measures of adult demographics and socioeconomic status come from the RAND HRS and include years of education, household income, and household wealth. Years of education is measured continuously as formal years of education and centered at its grand mean in analyses. Total household income is the sum of all income in a household including job earnings, pensions and annuities, social security or disability income, or other sources of income. Income is measured in nominal dollars and converted into 2000 dollars using the Consumer Price Index (CPI) in analyses. Total wealth is equal to the sum of all wealth components (e.g., value of first house, savings, accounts, investment accounts etc.) minus debt. Income and wealth are both centered at their grand mean and log-transformed to improve interpretability of the intercept and respective coefficient.

Several demographic variables potentially confound the relationship between socioeconomic status and weight, including race and ethnicity, nativity, and marital status. Race is trichotomized with white (reference category), black or other as the categories. Hispanic ethnicity is treated separately from race, and dichotomized (1 = Hispanic; 0 otherwise). Nativity is measured at baseline and dichotomized (1 = foreign born; 0 otherwise). Marital status is recoded into a series of dummy variables (married, separated or divorced, widowed, and never married). Age is also included as a control variable and measured in years.

#### Independent variables-neighborhood SES and the built environment

Neighborhood SES is measured using two previously validated scales [58] found to be significantly associated with obesity among older adults [42]. These scales reflect the economic advantage and disadvantage of a census tract. The economic advantage scale includes three variables: 1) the upper quartile value of owner-occupied housing units in the tract; 2) percentage of households with total annual income of $75 000 or more (2000 Census); and 3) percentage of adults with a college degree. The economic disadvantage scale includes six variables: 1) percentage of the total population in poverty; 2) percentage of the population 65 years and older in poverty; 3) percentage of households receiving public assistance income; 4) civilian unemployment rate among persons 16 years and older; 5) percentage of housing units without a vehicle; and 6) percentage black.

The built environment of the neighborhood census tract is measured using three variables: 1) population density, median age of neighborhood structures, and percentage of residents walking to work. These indicators have previously been used as measures of the built environment [59] and walkability [60]. All scales were constructed by summing transformed values (z-scores) and then standardized such that a one-unit change indicates a 1-standard deviation change. In addition to scales of economic advantage, disadvantage, and the built environment, three single-item measures of neighborhood characteristics were included to more fully capture the social environment of a neighborhood: residential stability (operationalized as percent of movers who lived in present house for at least one year), percent over the age of 65, and percent of population rural (2000 Census).

### Statistical Analysis

Body mass index and obesity were modeled as a function of individual and neighborhood characteristics using hierarchical linear models. To account for the clustering of individuals within census tracts, a random intercept model was estimated. In this analysis, level 1 variables measure individual characteristics and level 2 variables measure census tract characteristics. *Statistical Analysis Software* (SAS) (version 9.4) was used for all data management and PROC GLIMMIX was used for analyses. Significance tests for variation in BMI between tracts were conducted using the ‘covtest’ option in PROX GLIMMIX. In determining statistical significance, p<0.05 is used, but exact p-values are reported in the tables.

Given previous research on sex-specific associations between both individual SES and neighborhood SES and BMI [61], models were stratified by sex. Models were estimated in the following order: Model 1 is an unconditional means model estimating the overall mean BMI and variation in BMI that occurs between individuals and between census tracts; Model 2 adjusts for childhood conditions (i.e., childhood SES and childhood health) and demographic controls; Model 3 additionally adjusts for adult socioeconomic status; Model 4 additionally adjusts for neighborhood characteristics. Models 2–4 also adjust for wave of observation, race and ethnicity, age, nativity, and marital status. Descriptive statistics are presented in Tables 1 and 2, results predicting continuous BMI are presented in the manuscript (Tables 3–6), and results of the sensitivity analysis for models predicting the likelihood of obesity using hierarchical logistic regression are included in S1–S4 Tables. Pseudo R^2^, a measure of variation explained, is reported at each level of the analysis (i.e., how much variation in BMI *within* a census tract is explained by the model and how much variation in BMI *between* census tracts is explained).

**Table 1:** Descriptive Statistics of Individual Characteristics, HRS 2006–2008 (N = 13,545).

Variable	Males (N = 5,544)			Females (N = 8,001)		
	Mean	SD	Nmiss	Mean	SD	Nmiss
Body Mass Index	29.06	4.93	0	29.22	6.50	0
Obese (vs. not obese)	0.38	0.49	0	0.39	0.49	0
*Demographics*						
Age (Overall)	68.45	9.91	0	67.67	10.86	0
26–46 (n = 186)	0.01	0.09	-	0.02	0.13	-
47–66 (n = 5810)	0.41	0.49	-	0.44	0.50	-
67–86 (n = 7009)	0.55	0.50	-	0.50	0.50	-
86–104 (n = 540)	0.03	0.17	-	0.05	0.21	-
Foreign Born	0.09	0.29	8	0.11	0.31	4
Black	0.11	0.32	0	0.14	0.35	0
White	0.78	0.41	-	0.74	0.44	-
Hispanic	0.09	0.28	-	0.10	0.30	-
Other	0.01	0.12	-	0.02	0.13	-
Married	0.81	0.39	0	0.58	0.49	1
Widowed	0.08	0.27	-	0.27	0.44	-
Seperated or Divorced	0.09	0.28	-	0.13	0.33	-
Never Married	0.03	0.17	-	0.03	0.16	-
*Childhood Measures*						
Poor Childhood SES	0.33	0.47	73	0.29	0.45	108
Poor or Fair Health	0.04	0.20	3	0.05	0.22	1
Family Moved	0.20	0.40	43	0.18	0.38	33
*Mother’s Education*						
< High School	0.48	0.50	0	0.55	0.50	0
High School	0.31	0.46	-	0.25	0.43	-
> High School	0.11	0.32	-	0.12	0.32	-
Mother Educ. Missing	0.10	0.30	-	0.09	0.28	-
*Father’s Education*						
< High School	0.51	0.50	0	0.54	0.50	0
High School	0.22	0.42	-	0.20	0.40	-
> High School	0.13	0.33	-	0.12	0.32	-
Father's Educ. Missing	0.14	0.34	-	0.15	0.35	-
*Father’s Employment Status*						
Ever Unemployed	0.21	0.41	0	0.20	0.40	0
Absent	0.07	0.25	-	0.09	0.29	-
Employed	0.71	0.45	-	0.70	0.46	-
Missing	0.01	0.11	-	0.01	0.10	-
*Adult Socioeconomic Status*						
Household Income (log)	11.45	1.15	0	11.11	1.34	0
Household Wealth (log)	11.96	4.83	0	11.29	5.25	0
Education (yrs)	12.81	3.29	12	12.44	3.00	8

Notes. '-' value for Nmiss indicates dummy categories that share N missing values with first category listed.

The mean of binary 0/1 variables is presented and is a proportion of individuals codes as ‘1’.

**Table 2:** Descriptive Statistics of Census Tract Characteristics, ACS 2005–2009 (N = 4,127).

Variable	Mean	SD	Range	Nmiss
*Socioeconomic Advantage*				
Housing Value Upper Quartile	286488.00	205331.00	3190–1000001	26
% Income Over $75K (HH)	0.20	0.15	0–0.86	1
% College Degree or More (I)	0.25	0.16	0–0.92	2
*Socioeconomic Disadvantage*				
% Below Poverty (I)	0.14	0.11	0–0.81	3
% Below Poverty, Over 65, (I)	0.10	0.14	0–0.23	3
% Receiving Public Income Assistance, (HH)	0.03	0.03	0–0.27	3
% Unemployed, (I)	0.05	0.03	0–0.29	3
%Without Vehicle, (HH)	0.08	0.11	0–0.89	3
% Black	0.14	0.24	0–1	2
*Built Environment and Demographics*				
Population Density (I per square mile)	4124.00	11478.74	0.5–208518	2
Structure Age	37.76	15.74	0–70	49
% Walk to Work	0.02	0.03	0–0.50	3
% Same Household From Previous Year (I)	0.85	0.08	0–1	2
% Over 65	0.15	0.08	0–1	2
% Rural	0.24	0.37	0–1	0

Notes. (HH) and (I) indicate household and individual, respectively.

**Table 3:** Hierarchical Linear Model Results for Female BMI, HRS 2006–2008.

Variables	Model 1		Model 2		Model 3		Model 4	
	β(SE)	p	β(SE)	p	β(SE)	p	β(SE)	p
Intercept	29.06(0.10)	<0.0001	27.15(0.26)	<0.0001	28.05(0.28)	<0.0001	28.22(0.29)	<0.0001
**Childhood Conditions**								
Financial Hardship			0.08(0.17)	0.6487	-0.02(0.17)	0.8915	-0.07(0.17)	0.6930
Poor or Fair Health			-0.05(0.33)	0.8860	-0.13(0.33)	0.6993	-0.13(0.33)	0.6885
Family Moved			0.08(0.20)	0.6979	0.01(0.20)	0.9716	0.01(0.20)	0.9721
**Mother’s Education**								
> High School (ref)								
< High School			0.37(0.27)	0.1655	0.03(0.27)	0.9011	-0.03(0.27)	0.9190
High School			0.02(0.26)	0.9258	-0.18(0.26)	0.5021	-0.17(0.26)	0.5096
Mother Educ. Missing			0.59(0.38)	0.1196	0.10(0.38)	0.7884	0.05(0.38)	0.8980
**Father’s Education**								
> High School (ref)								
< High School			1.23(0.27)	<0.0001	0.91(0.27)	0.0008	0.68(0.27)	0.0137
High School			1.01(0.28)	0.0002	0.83(0.28)	0.0026	0.67(0.28)	0.0149
Father's Educ. Missing			1.37(0.34)	<0.0001	0.93(0.35)	0.0076	0.70(0.35)	0.0446
**Father's Employment**								
Employed (ref)								
Unemployed			0.02(0.19)	0.9025	0.01(0.19)	0.9626	0.02(0.19)	0.9306
Absent			-0.43(0.28)	0.1228	-0.51(0.28)	0.0652	-0.50(0.28)	0.0739
Missing Data			-0.00(0.73)	0.9981	-0.00(0.73)	0.9957	0.01(0.73)	0.9839
**Adult Socioeconomic Status**								
Household Income (log)					-0.14(0.06)	0.0327	-0.07(0.06)	0.2557
Household Wealth (log)					-0.08(0.02)	<0.0001	-0.07(0.02)	<0.0001
Education (years)					-0.15(0.03)	<0.0001	-0.11(0.03)	0.0003
**Neighborhood Characteristics**							
SES Advantage							-0.62(0.09)	<0.0001
SES Disadvantage							-0.03(0.11)	0.7674
Built Environment							-0.01(0.09)	0.9261
% Same Household							0.17(0.08)	0.0361
% Over 65							-0.10(0.07)	0.1651
% Rural							0.09(0.08)	0.2930

Notes. Models 2–4 include controls for wave of interview, age, race and ethnicity, nativity, and marital status.

**Table 4:** Variance Components and Model Fit Statistics, Females, HRS 2006–2008.

	Model 1		Model 2		Model 3		Model 4	
Variance Components		p		p		p		p
Between census tract	1.58(0.46)	<0.0001	0.96(0.41)	0.0037	0.76(0.39)	0.0148	0.56(0.37)	0.0501
Within census tract	40.62(0.76)	<0.0001	38.34(0.72)	<0.0001	38.13(0.71)	<0.0001	38.02(0.70)	<0.0001
Pseudo *R*^2^ and Goodness of Fit								
*R*^2^, Between Tract	-		0.39		0.52		0.65	
*R*^2^, Within tract	-		0.06		0.06		0.06	
-2Log Likelihood	52636.14		51145.02		51031.78		50710.1	
AIC	52640.14		51149.02		51035.78		50714.1	
N, Level 2	4127		4077		4074		4039	
N, Level 1	8001		7857		7850		7808	

**Table 5:** Hierarchical Linear Model Results for Male BMI, HRS 2006–2008.

Variables	Model 1		Model 2		Model 3		Model 4	
	β(SE)	p	β(SE)	p	β(SE)	p	β(SE)	p
Intercept	29.05(0.07)	<0.0001	28.57	<0.0001	28.63(0.25)	<0.0001	28.77(0.25)	<0.0001
**Childhood Conditions**								
Financial Hardship			0.35(0.16)	0.0265	0.34(0.16)	0.0333	0.32(0.16)	0.0424
Poor or Fair Health			-0.58(0.34)	0.0874	-0.58(0.34)	0.0858	-0.61(0.34)	0.0727
Family Moved			0.06(0.18)	0.7397	0.04(0.18)	0.8256	0.04(0.18)	0.8362
**Mother’s Education**								
> High School (ref)								
< High School			0.22(0.26)	0.3938	0.15(0.26)	0.5588	0.07(0.26)	0.7888
High School			0.45(0.24)	0.0651	0.41(0.24)	0.0899	0.36(0.24)	0.1388
Mother Educ. Missing			-0.07(0.35)	0.8444	-0.18(0.35)	0.6095	-0.26(0.36)	0.4740
**Father’s Education**								
> High School (ref)								
< High School			0.30(0.25)	0.2358	0.27(0.25)	0.2786	0.16(0.25)	0.5320
High School			0.00(0.25)	0.9956	-0.00(0.25)	0.9904	-0.11(0.25)	0.6537
Father's Educ. Missing			-0.01(0.33)	0.9688	-0.06(0.33)	0.8677	-0.15(0.33)	0.6630
**Father's Employment**								
Employed (ref)								
Unemployed			0.36(0.18)	0.0428	0.36(0.18)	0.0413	0.37(0.18)	0.0378
Absent			0.50(0.29)	0.0892	0.49(0.29)	0.0939	0.50(0.29)	0.0913
Missing Data			0.23(0.71)	0.7436	0.37(0.72)	0.6059	0.37(0.73)	0.6116
**Adult Socioeconomic Status**								
Household Income (log)					0.19(0.07)	0.0062	0.22(0.07)	0.0013
Household Wealth (log)					-0.02(0.02)	0.2994	-0.01(0.02)	0.3893
Education (years)					-0.06(0.03)	0.0262	-0.03(0.03)	0.1842
**Neighborhood Characteristics**								
SES Advantage							-0.43(0.09)	<0.0001
SES Disadvantage							-0.18(0.11)	0.0972
Built Environment							0.04(0.08)	0.6520
% Same Household							0.17(0.08)	0.0232
% Over 65							-0.02(0.07)	0.7686
% Rural							-0.12(0.08)	0.7686

Notes. Models 2–4 include controls for wave of interview, age, race and ethnicity, nativity, and marital status.

**Table 6:** Variance Components and Model Fit Statistics, HRS 2006–2008.

	Model 1		Model 2		Model 3		Model 4	
Variance Components		p		p		p		P	
Between Tract	0.68(0.33)	0.0118	0.54(0.33)	0.0379	0.53(0.33)	0.0434	0.47(0.33)	0.0646	
Within Tract	23.63(0.54)	<0.0001	23.10	<0.0001	23.11(0.54)	<0.0001	23.10(0.55)	
Pseudo *R*^2^ and Goodness of Fit								
*R*^2^, Between Tract	-		0.21		0.22		0.31	
*R*^2^, Within Tract	-		0.02		0.02		0.02	
-2Log Likelihood	33421.31		32541.76		32496.69		32327.17	
AIC	33425.31		32545.76		32500.67		32331.17	
N, Level 2	3124		3077		3074		3046	
N, Level 1	5544		5422		5412		5384	

#### Centering decisions

An inherent difficulty in multilevel modeling is the attempt to summarize the association between two variables using a single regression parameter because the association may in fact be due to variation at multiple levels of hierarchy [62]. For example, an observed association between childhood health and adult BMI in a multilevel analysis that includes measures at level 1 (individuals) and level 2 (census tracts) may be due to variation in individual childhood health as well as variation in mean childhood health between census tracts.

One approach to deal with this problem is to center level 1 observations at their group mean (e.g., center an individual’s childhood health score at the mean of the census tract), thereby removing all variation in the independent variable due to level 2 variation. Doing so assures that estimates of an association between a level 1 variable and the outcome of interest are independent from level 2 variables, hence group-mean centering is recommended when the parameter of interest involves a level 1 predictor [63]. Although the present research is interested in childhood conditions as measured by a series of level 1 variables, group-mean centering would result in level 1 predictors orthogonal to level 2 predictors, guaranteeing that the association between childhood conditions and adult BMI would remain unaffected by the inclusion of level 2 variables. Childhood conditions are therefore left in their raw metric (dummy coded) in the analysis to preserve any correlation they may have with census tract characteristics. Interpretation of the association between childhood conditions and adult BMI is thus the same as in a single-level model, with one important caveat: the association may be a blend of level 1 and level 2 variation in childhood conditions. Furthermore, because neighborhood characteristics can only correlate with the between-cluster variation in childhood conditions, the association between childhood conditions and adult BMI can only be “explained” by census tract characteristics if the association is due entirely to between-tract variation in childhood conditions. As will be discussed in the results section, only a small proportion of variation in BMI is due to variation in mean BMI between tracts, making it unlikely that census tract characteristics can fully account for the association between childhood conditions and adult BMI. Level-1 measures of age, education, household income, and household wealth are grand-mean centered. Centering decisions at level 2 are somewhat easier and follow the general recommendations of standard OLS regression [62]. Level 2 variables are centered at their grand mean to improve interpretability of the intercept. Number of observations per census tract ranged from 1–48, with 44% of census tracts having a single observation. The detrimental impact of singletons on multilevel point and interval estimates decreases as the sample of level-2 observations increases, and appears to be negligible with a level-2 N greater than 500 [64]. Given the large number of level-2 observations in the analysis (N = 4,127), point and interval estimates in the present analysis are unlikely to be affected by singletons.

## Results

Table 1 reports descriptive statistics for individual characteristics stratified by sex. Among females, mean BMI and age are 29.22 kg/m^2^ and 67.67 years, respectively, and 39% are obese. Among males, mean BMI and age are 29.06 kg/m^2^ and 67.7 years, respectively, and 39% are obese. Table 2 reports descriptive statistics for census tracts. Regarding measures of neighborhood socioeconomic advantage (at the census tract level), the mean upper quartile housing value is $286,488, with an average of 20% of households having an income over $75,000 and 25% of individuals possessing a college degree or more. Regarding socioeconomic disadvantage, mean percent of individuals below the poverty line is 14%. Average structure age is 37.8 years. Tables 3 and 4 report estimated fixed effects and model fit statistics/variance estimates, respectively, for females. Among females in Model 1 (Table 3), the unconditional means model estimates an overall mean BMI of 29.06 (p<0.05). A small but significant proportion of variation in BMI is attributable to differences between tracts (σ^2^_0_ = 1.58, p<0.05), while a much larger proportion of variation in BMI is attributable to differences between individuals (σ^2^_ε_ = 40.62, p<0.05). The proportion of BMI variation attributable to differences in mean BMI between tracts (ρ) is equal to σ^2^_0_ / (σ^2^_0_+ σ^2^_ε_) = 3.7% (Table 4). As even small intra-class correlation coefficients, such as what is observed for females (and males), can impact estimates of standard errors and increase the probability of Type 1 errors, we retain a hierarchical analysis in subsequent models [65].

Model 2 (Table 3) for females includes childhood conditions and all control variables. Relative to fathers with a college degree or more, having a father with less than a high school degree (b = 1.23, p<0.05) or a high school degree (b = 1.01, p<0.05) is associated with a higher BMI. Other measures of childhood conditions were not significantly associated with BMI. Individual control variables and childhood conditions explain 39% of the variation in BMI between census tracts and 6% of the variation within census tracts (Table 4). Model 3 (Table 3) additionally adjusts for adult socioeconomic status. Household income (b = -0.14, p<0.05), household wealth (b = -0.08, p<0.05) and years of education (b = -0.15, p<0.05) were all inversely associated with BMI. After adjusting for adult SES, father’s education remains significantly associated with BMI: b(less than high school) = 0.91, p<0.05), b(high school) = 0.83, p<0.05). Adult SES explains an additional 13% of the variation in BMI between census tracts but <1% additional variation within census tracts. Model 4 (Table 3) additionally adjusts for neighborhood characteristics. A higher score on the index of neighborhood SES advantage was significantly associated with lower BMI (b = -0.62, p<0.01), while living in a neighborhood with a greater percent of individuals who had not moved in the previous year was associated with higher BMI (b = 0.17, p<0.05). After adjusting for neighborhood characteristics, father’s education remains significantly associated with adult female BMI: b(less than high school) = 0.68, p<0.05), b(high school) = 0.67, p<0.05).Measures of neighborhood characteristics explain an additional 13% of the variation in BMI between census tracts.

Tables 5 and 6 report estimated fixed effects and model fit statistics/variance estimates, respectively, for males. Model 1, the unconditional means model, estimates an overall mean BMI of 29.05 (p<0.05). As with females, a small but significant proportion of variation in BMI is attributable to differences between tracts (σ^2^_0_ = 0.68, p<0.05), while a much larger proportion of variation in BMI is attributable to differences between individuals (σ^2^_ε_ = 23.63, p<0.05). Among men, the proportion of BMI variation attributable to differences in mean BMI between tracts is 2.8%.

Model 2 (Table 5) includes childhood conditions and demographic controls. Two indicators of childhood SES were associated with BMI: perceived financial status of the household and father’s employment. Individuals who reported living in a household with poor SES as a child were predicted to have a higher BMI as an adult (b = 0.35, p<0.05), as was having a father who was at one point unemployed (b = 0.36, p<0.05). Other measures of childhood conditions were not significantly associated with BMI. Individual control variables and childhood conditions explain 21% of the variation in BMI between census tracts and 2% of the variation within census tracts (Table 6).

Model 3 (Table 5) additionally adjusts for adult socioeconomic status. Household income was positively associated with BMI (b = 0.19, p<0.05), while years of education was negatively associated with BMI (b = -0.06, p<0.05). After adjusting for adult SES, perceived financial hardship as a child and having an unemployed father remained significantly associated with a higher BMI. Adult SES explains an additional 1% of the variation in BMI between census tracts but <1% additional variation within census tracts.

Model 4 additionally adjusts for neighborhood characteristics. Living in a socioeconomically advantaged census tract was associated with a lower BMI (b = -0.43, p<0.01), while living in an area where a higher percentage of residential stability was associated with a higher BMI (b = 0.17, p<0.05). After adjusting for neighborhood characteristics, financial hardship as a child and having an unemployed father remained associated with adult BMI. Measures of neighborhood characteristics explain an additional 9% of the variation in BMI between census tracts.

While results from models with a continuous measure of BMI indicate a significant association between some indicators of childhood conditions and adult BMI, the above findings must take into consideration the null results from sensitivity tests: in models estimating the likelihood of obesity rather than a continuous measures of BMI, no indicator of childhood socioeconomic status was associated with the likelihood of obesity after adjusting for neighborhood SES in females and after adjusting for demographics in males. Because no indicator of childhood conditions was associated with obesity among males after controlling for demographics, models that additionally adjust for individual adult SES and neighborhood SES for males were not estimated.

## Discussion

This analysis tested whether childhood conditions, as measured by several indicators of childhood SES and childhood health, are independently associated with adult BMI after adjusting for individual and contextual measures of adult socioeconomic status.

Previous research has found an independent association between various indicators of childhood SES and adult BMI after accounting for adult SES [14], yet little research has included measures of neighborhood characteristics as an additional measure of adult SES. The novelty of this research is therefore its synthesis of life course and ecological approaches to the examination of early life conditions and adult weight, ultimately providing a stronger test of a critical period model linking childhood conditions to adult weight.

Results from this analysis suggest that some indicators of childhood SES, namely father’s education among females, and father’s occupation and perceived financial hardship among males, are independently associated with adult BMI after accounting for both individual and contextual measures of socioeconomic status, supporting a critical period model for these specific childhood conditions. Among females, having a father with less than a college degree was associated with increased BMI as an adult. Overall, 39% and 6% of the variation in BMI among females *between* census tracts and *within* census tracts, respectively, was associated with childhood conditions and demographic controls. Adult SES explained an additional 13% of the variation in BMI between census tracts, and adult neighborhood SES a further 13%. Among males, having an unemployed father and perceived financial hardship as a child were associated with increased adult BMI. Overall, 21% and 2% of the variation in BMI among males between census tracts and within census tracts, respectively, was associated with childhood conditions and demographic controls. Adult SES explained an additional 1% of the variation in BMI between census tracts, and adult neighborhood SES a further 9%.

Findings from the present analysis are broadly similar to previous research on childhood conditions and adult health, but not uniformly so. Childhood health was not associated with adult BMI or obesity among males or females, contrasting with prior research on the influence of childhood health and adult outcomes, which has indicated poor childhood health to be associated with worse employment outcomes and health as an adult [66–69]. Other indicators of childhood health, namely birth weight and birth length, may be better predictors of adult BMI or obesity than the measures used in the HRS (a retrospective measure of self-reported health) [70, 71]. The findings for father’s education among females are consistent with Best and Hayward et al 2005 [72], who found an inverse association between father’s education and female obesity BMI (but not male obesity) using data from the HRS. In the present analysis, among females, other indicators of childhood conditions, including financial hardship and childhood health, did not exhibit a pattern consistent with either a critical period model or accumulation of risk model, as none were significantly associated with adult BMI in models with demographic controls. Among males, financial hardship and father’s employment pattern were associated with adult BMI after controls for individual and neighborhood SES as an adult, consistent with a critical period model. Among males, as with females, no other indicators of childhood conditions exhibited a pattern consistent with either a critical period model or accumulation of risk model, as none were significantly associated with adult BMI in models with demographic controls.

The medical and health relevance of the above results using a continuous measure of BMI must be viewed in the context of the results of the sensitivity analysis with BMI dichotomized as obese vs. not obese. Among females, having a father with less than a high school degree was associated with a greater likelihood of obesity even after adjusting for adult SES; however, the association was not significant after adjusting for neighborhood characteristics. This suggests that among females, the influence of paternal education on obesity specifically may be more consistent with an accumulation of risk model. Among males, no association between childhood conditions and likelihood of obesity was observed in models adjusted for demographics. While these results do not contradict analyses using a continuous measure of BMI, they may indicate that in a population where the majority of older adults are overweight or obese [3], coming from a higher SES household of origin does not exert a sufficient effect to counteract other environmental exposures, either in early or later life, to reduce the risk of obesity independent from adult SES and neighborhood exposures. Furthermore, while the present analysis provides evidence of an independent association between some indicators of childhood SES and adult weight, consistent with a critical period model, these indicators may not be independent from adult health behaviors (e.g., smoking. Finally, the lack of a significant association between indicators of childhood conditions in models examining the likelihood of being obese versus not obese may be due to the loss of statistical power that often accompanies dichotomizing quantitative variables [73].

Among measures of neighborhood characteristics, results indicated that living in an economically advantaged tract was associated with a lower BMI among males and females, while residing in an economically disadvantaged tract was not associated with BMI. The findings for economic advantage are consistent with Grafova et al.’s [42] analysis of neighborhood characteristics among older adults using self-reported height and weight. The effect on weight of residing in an economically advantaged tract may be due to a combination of material and psychosocial resources, cultural influences, or selection, but these possibilities cannot be disentangled in this analysis. Residential stability was also associated with BMI—residing in a tract with a higher percentage of residents who had not moved in the previous year was associated with a higher BMI, consistent with the findings of Grafova, Freedman (42). Residential stability in the past year may indicate greater duration of exposure to the environmental characteristics of one’s neighborhood, but moving in the past year may also represent a neighborhood where a significant number of individuals are healthy enough to have recently moved which, based on the results of this analysis, may include individuals with a lower BMI. Additionally, residential stability may be positively correlated with age and rurality, both of which are positively associated with BMI [3, 74].

As indicated previously, findings are consistent with a model of direct childhood for some indicators of childhood SES—paternal education among females and paternal employment and financial hardship among males. Though important, paternal education and employment are but two of many other early influences on later weight gain. A review of reviews identified several early risk-factors for later obesity, including infant obesity, maternal diabetes, maternal smoking, and infrequent physical activity [75]. Among early life risk factors, Monasta et al. (2010) also included low parental socioeconomic status as an early risk factor for subsequent weight gain, though they concluded the evidence for its association was weaker. Overall, early childhood SES is but one factor among many that social scientists and practitioners should consider in their efforts to understand and influence adult weight.

Findings from the present research suggest sex differences in the influence of childhood socioeconomic status. Why might differences be observed? One possibility is that the effects of some childhood conditions may be mediated by physiological sex differences, such as the increased production of testosterone and increased lean mass in pubescent boys, [76] altering long-term trajectories of weight gain and mitigating obesity-promoting risks of pre-pubescent exposures. A second possibility is that early exposure to an economically advantaged background magnifies gender differences in socialization regarding ideal weight and body shape. Ogden [77] found that girls from a higher class reported greater body dissatisfaction, concern about weight, and restrained eating behaviors. It may be that girls raised in households with greater paternal education are exposed to an aesthetic that emphasizes thinness, exerting a lifelong influence on eating and exercise behaviors. Among males, this aesthetic may not be as emphasized in higher SES households as indicated by paternal education; however, coming from a household with an unemployed father or perceived financial hardship may indicate greater material disadvantage, ultimately leading to increased BMI as an adult. It is important to note that while the estimated effect of paternal education is significantly different from zero among females but not among males, this does not indicate that the estimated effect for females is significantly different from the estimated effect of males, as such a conclusion would require a separate set of statistical tests comparing the coefficients to each other rather than each coefficient to zero. The argument that differences in socialization by class may explain differences in BMI as an adult parallels evidence that body aesthetics may also vary by race and ethnicity. Some evidence suggests that African Americans perceive a larger body size as ideal relative to Americans of European descent [78, 79]. Future research may wish to examine the competing influences of class and ethnicity on beliefs about the ideal body size.

The potential lifelong influence of early exposure to cultural values on health, (perhaps stronger among females with a more educated father), parallels the logic of the critical period model [80]. Although biological scarring is a commonly identified mechanism linking critical period exposures to subsequent disease outcomes, Kuh and Ben-Schlomo et al. (2003) do not limit critical period effects to such mechanisms. Critical periods may also exist for the acquisition of norms or behavior. Language acquisition is a useful analogy, as language acquisition has long been hypothesized to have a critical period of development, whereby it is easier to learn a language between the ages of five and puberty [81]. Likewise, there may be a critical period of development for the acquisition and internalization of beauty norms. Although exposure to cultural notions regarding ideal body size and beauty occurs across the life course, exposure to cultural beliefs about the body early in life may engender a cultural fluency with lifelong implications for weight related health outcomes.

An additional issue in the interpretation of paternal education’s association with lower adult BMI among females is the changing nature and availability of higher education in the US. Children born between 1931 and 1941 were born in an era of significantly lower levels of educational attainment. In 1940, only 25% of the population had completed high school. But by 2007, 87% had completed high school [82, 83]. A similarly dramatic increase occurred in the percent of US adults with a college degree—in 1940 only 5% of the population had a college degree, but by 2007 the percentage had increased to 28% (higher than the percentage of adults with a high school degree in 1940). Such a low level of educational attainment during this era is important given that the parents of the many HRS participants were school-age at the time. The meaning of education has surely changed from a time when college attendance was rare and national magazines published articles trying to convince a skeptical nation that a woman could simultaneously be attractive and go to college [84]. Insofar as educational institutions serve to reinforce cultural notions of beauty, as notions of beauty change, so too should the effect of education on adult weight. Thus, the findings of this analysis may not generalize to younger cohorts. Indeed, Reither, Hauser [85] found that the 1990s marked a change in the previously step-wise association between education and obesity; whereas prior to the 1990s “each step up the educational ladder” was associated with a decline in the likelihood of obesity, only a college degree was associated with a reduced likelihood of obesity beginning in the late 1990s.

There are several strengths of this study—notably the use of technician measured height and weight and a comprehensive set of measures for both childhood conditions and adult SES. Importantly, measures of adult SES included neighborhood characteristics. To date, most life-course research linking early life-conditions to adult weight has focused only on individual SES as a potential mediator between early life and subsequent weight. The present research, by incorporating adult neighborhood SES in its analysis, synthesizes life-course and ecological perspectives, providing a more powerful test of childhood SES as an independent predictor of adult weight.

There are also important limitations to this research in addition to the limitations of a multilevel analysis previously discussed. First, the lack of longitudinal measures of neighborhood exposure is a significant limitation of this research, which may underestimate its influence on health [86]. Due to limited data availability for technician measured height and weight, demographic and socioeconomic information of census tracts only reflect estimated census tract characteristics for 2006 and 2008. Prior to 2006, only self-reported height and weight is available in the HRS. A second limitation is the lack of measured obesity early in life for the HRS respondents, which may lead to overestimates of the association between other childhood characteristics and adult BMI. A 2008 systematic review on the tracking of overweight and obesity from childhood to adulthood found that all studies reported a consistently positive link between childhood overweight and obesity and adult overweight and obesity status [87]. Physiological maternal characteristics are also unavailable in the HRS, though they have important influences on fetal development, early life adiposity, and adult weight [27].

The use of retrospective measures of childhood conditions is another limitation. Some research has cast doubt on the validity of recall measures with regards to adverse childhood experiences, including sexual or physical abuse [88], but other research is more optimistic about the use of retrospective measures of childhood socioeconomic position [89]. Regarding measures of childhood health, research has found that recall measures of childhood health in the Health and Retirement Study to be reliable, although there is no method to determine the validity of this measure because the HRS did not directly measure childhood health [90].

The use of census tracts as an indicator for neighborhood residence may not fully capture the local area in which an individual spends most of their time. Although the use of census tracts is standard practice in much of the literature on neighborhood characteristics and health, census tracts may be too rough a proxy for neighborhoods to capture sufficient variation in BMI given the limited between-tract variation in BMI observed in the present analysis. Finally, mortality prior to the 2006–2008 has the potential to affect the observed association between childhood conditions and weight. Excess mortality has been observed in both underweight populations and obese populations, with greater excess observed in higher degrees of obesity [91]. Consequently, the largest population on which this study was based—those surviving to middle age—likely has a low representation of those who were underweight or obese compared to earlier ages. Less clear is the mortality risk associated with being overweight relative to those classified as having a healthy BMI. Some research has found that being overweight is associated with reduced mortality risk [92, 93]. If childhood conditions are associated with an increased risk of more extreme obesity, analyses may underestimate the association because obese individuals will not be included in the analysis. If selection due to mortality differs by sex, then observed sex differences in the association between childhood conditions and adult weight may be due to differential mortality, and any conclusions about sex differences in the relationship between childhood conditions and mortality are more tentative than conclusions about the estimated associations within a sex category, which are likely to be conservative among obese individuals.

To conclude, results suggest that childhood SES is associated with adult BMI (but not BMI category) after adjusting for adult individual and neighborhood SES. These findings strengthen the existing body of evidence that early life social conditions matter for adult weight. Though several mechanisms have been proposed in this paper for the long-term association between childhood SES and adult weight, including the modeling of health behaviors by parents and the socialization of beauty norms among females, the exact mechanisms remain unknown. Future research should more precisely identify the timing of the influence childhood SES and its relative contribution to adult body weight in relation to other early life experiences (e.g., maternal factors), which requires the synthesis of sociological, physiological, and psychological perspectives of human development.

## Supporting Information

S1 Table Hierarchical Logistic Regression Results Estimating Likelihood of Obesity among Females, HRS2006-2008. (DOCX)

S2 Table Variance Components and Model Fit Statistics, HRS 2006–2008. (DOCX)

S3 Table Hierarchical Logistic Regression Results Estimating Likelihood of Obesity among Females, HRS2006-2008. (DOCX)

S4 Table Variance Components and Model Fit Statistics, HRS 2006–2008. (DOCX)
