# Supplementary material for: Is Childhood Socioeconomic Status Independently Associated with Adult BMI after Accounting for Adult and Neighborhood Socioeconomic Status?
Source: PLoS One. 2017 Jan 17;12(1):e0168481. doi: 10.1371/journal.pone.0168481 (PMC5241009; doi:10.1371/journal.pone.0168481)
Supplement: S1 Table — (DOCX) [file pone.0168481.s001.docx]

S1 Table. Hierarchical Logistic Regression Results Estimating Likelihood of Obesity among Females, HRS2006-2008

|  | | | | | | | |  |
| --- | --- | --- | --- | --- | --- | --- | --- | --- |
| Variables | Model 1 |  | Model 2 |  | Model 3 |  | Model 4 |  |
|  | OR (95% CI) | p | OR (95% CI) | p | OR (95% CI) | p | OR (95% CI) | p |
| Intercept (Logit) | -0.47 | <0.0001 | -1.09 | <0.0001 | -0.83 | <0.0001 | -0.78 | <0.0001 |
| **Childhood Conditions** |  |  |  |  |  |  |  |  |
| Financial Hardship |  |  | 1.04(0.93,1.17) | 0.4623 | 1.01(0.90,1.14) | 0.8082 | 1.01(0.90,1.13) | 0.1208 |
| Poor or Fair Health |  |  | 0.93(0.75,1.16) | 0.5314 | 0.91(0.73,1.14) | 0.4035 | 0.92(0.74,1.15) | 0.4556 |
| Family Moved |  |  | 0.96(0.84,1.10) | 0.5752 | 0.94(0.82,1.08) | 0.3659 | 0.93(0.82,1.07) | 0.3220 |
| **Mother’s Education** |  |  |  |  |  |  |  |  |
| > High School (ref) |  |  |  |  |  |  |  |  |
| < High School |  |  | 1.22(1.02,1.47) | 0.0339 | 1.10(0.91,1.32) | 0.3236 | 1.07(0.89,1.30) | 0.4461 |
| High School |  |  | 1.12(0.93,1.33) | 0.2262 | 1.05(0.88,1.26) | 0.5939 | 1.04(0.87,1.25) | 0.6462 |
| Mother Educ. Missing |  |  | 1.31(1.02,1.69) | 0.0355 | 1.14(0.88,1.47) | 0.3314 | 1.11(0.86,1.43) | 0.4431 |
| **Father’s Education** |  |  |  |  |  |  |  |  |
| > High School (ref) |  |  |  |  |  |  |  |  |
| < High School |  |  | 1.35(1.13,1.63) | 0.0012 | 1.24(1.03,1.50) | 0.0232 | 1.16(0.97,1.40) | 0.1136 |
| High School |  |  | 1.25(1.04,1.51) | 0.0186 | 1.19(0.99,1.44) | 0.0702 | 1.14(0.95,1.38) | 0.1645 |
| Father's Educ. Missing |  |  | 1.33(1.05,1.68) | 0.0163 | 1.18(0.93,1.49) | 0.1795 | 1.10(0.87,1.40) | 0.4156 |
| **Father's Employment** |  |  |  |  |  |  |  |  |
| Employed (ref) |  |  |  |  |  |  |  |  |
| Unemployed |  |  | 1.01(0.89,1.15) | 0.8675 | 1.01(0.89,1.15) | 0.9026 | 1.01(0.89,1.15) | 0.8644 |
| Absent |  |  | 0.90(0.74,1.08) | 0.2511 | 0.87(0.72,1.05) | 0.1590 | 0.87(0.72,1.05) | 0.1560 |
| Missing Data |  |  | 0.92(0.57,1.50) | 0.7489 | 0.92(0.57,1.50) | 0.7417 | 0.95(0.58,1.54) | 0.8239 |
| **Adult Socioeconomic Status** |  |  |  |  |  |  |  |  |
| Household Income (log) |  |  |  |  | 0.96(0.92,1.01) | 0.0875 | 0.98(0.94,1.02) | 0.3384 |
| Household Wealth (log) |  |  |  |  | 0.98(0.97,0.99) | <0.0001 | 0.98(0.97,0.99) | <0.0001 |
| Education (years) |  |  |  |  | 0.96(0.94,0.98) | <0.0001 | 0.97(0.95,0.99) | 0.0016 |
| **Neighborhood Characteristics** | |  |  |  |  |  |  |  |
| SES Advantage |  |  |  |  |  |  | 0.84(0.79,0.90) | <0.0001 |
| SES Disadvantage |  |  |  |  |  |  | 0.98(0.91,1.05) | 0.5412 |
| Built Environment |  |  |  |  |  |  | 1.01(0.96,1.07) | 0.6866 |
| % Same Household |  |  |  |  |  |  | 1.03(0.98,1.09) | 0.3335 |
| % Over 65 |  |  |  |  |  |  | 0.98(0.93,1.03) | 0.4206 |
| % Rural |  |  |  |  |  |  | 1.03(0.98,1.10) | 0.2577 |
|  | | | | | | |  |  |

Notes. Models 2-4 include controls for wave of interview, age, race and ethnicity, nativity, and martial status.
