# Supplementary material for: Is Childhood Socioeconomic Status Independently Associated with Adult BMI after Accounting for Adult and Neighborhood Socioeconomic Status?
Source: PLoS One. 2017 Jan 17;12(1):e0168481. doi: 10.1371/journal.pone.0168481 (PMC5241009; doi:10.1371/journal.pone.0168481)
Supplement: S2 Table — (DOCX) [file pone.0168481.s002.docx]

|  | | | | | |  |  |  |
| --- | --- | --- | --- | --- | --- | --- | --- | --- |
|  | Model 1 | | Model 2 | | Model 3 | | Model 4 | |
| Variance Components | | p |  | p |  | p |  | p |
| Between Tract | 0.22(0.39) | 0.0005 | 0.07(0.04) | 0.0158 | 0.06(0.04) | 0.0389 | 0.04(.04) | 0.1042 |
| -2Log Likelihood | 10729.91 |  | 10143.21 |  | 10079.7 |  | 9986.85 |  |
| Pseudo *R*^2^ and Goodness of Fit |  |  |  |  |  |  |  |  |
| *R*^2^, Between Tract | - |  | 0.68 |  | 0.72 |  | 0.82 |  |
| AIC | 10735.91 |  | 10189.21 |  | 10129.7 |  | 10048.85 |  |
| N, Level 2 | 4127 |  | 4077 |  | 4074 |  | 4039 |  |
| N, Level 1 | 8001 |  | 7857 |  | 7850 |  | 7808 |  |

S2 Table. Variance Components and Model Fit Statistica, HRS 2006-2008.
