# Supplementary material for: Is Childhood Socioeconomic Status Independently Associated with Adult BMI after Accounting for Adult and Neighborhood Socioeconomic Status?
Source: PLoS One. 2017 Jan 17;12(1):e0168481. doi: 10.1371/journal.pone.0168481 (PMC5241009; doi:10.1371/journal.pone.0168481)
Supplement: S3 Table — (DOCX) [file pone.0168481.s003.docx]

S3 Table. Hierarchical Logistic Regression Results Estimating Likelihood of Obesity among Males, HRS 2006-2008.

| Variables | Model 1 |  | Model 2 |  | Model 3 |  | Model 4 |  |
| --- | --- | --- | --- | --- | --- | --- | --- | --- |
|  | OR (95% CI) | p | OR (95% CI) | p | OR (95% CI) | p | OR (95% CI) | p |
| Intercept (Logit) | -0.53(0.04( | <0.0001 | -0.77(0.10) | <0.0001 |  |  |  |  |
| **Childhood Conditions** |  |  |  |  |  |  |  |  |
| Financial Hardship |  |  | 1.09(0.96,1.25) | 0.1912 | - |  | - |  |
| Poor or Fair Health |  |  | 0.90(0.68,1.21) | 0.4898 | - |  | - |  |
| Family Moved |  |  | 1.07(0.92,1.25) | 0.3686 | - |  | - |  |
| *Mother’s Education* |  |  |  |  |  |  |  |  |
| > High School (ref) |  |  |  |  |  |  |  |  |
| < High School |  |  | 1.10(0.88,1.36) | 0.4190 | - |  | - |  |
| High School |  |  | 1.17(0.95,1.44) | 0.1347 | - |  | - |  |
| Mother Educ. Missing |  |  | 1.16(0.86,1.56) | 0.3450 | - |  | - |  |
| **Father’s Education** |  |  |  |  |  |  |  |  |
| > High School (ref) |  |  |  |  |  |  |  |  |
| < High School |  |  | 1.16(0.94,1.44) | 0.1585 | - |  | - |  |
| High School |  |  | 1.07(0.86,1.31) | 0.5552 | - |  | - |  |
| Father's Educ. Missing |  |  | 1.01(0.76,1.34) | 0.9538 | - |  | - |  |
| **Father's Employment** |  |  |  |  |  |  |  |  |
| Employed (ref) |  |  |  |  |  |  |  |  |
| Unemployed |  |  | 1.14(0.98,1.32) | 0.0936 | - |  | - |  |
| Absent |  |  | 1.10(0.86,1.41) | 0.4619 | - |  | - |  |
| Missing Data |  |  | 0.92(0.50,1.70) | 0.7936 | - |  | - |  |
| **Adult Socioeconomic Status** |  |  |  |  |  |  |  |  |
| Household Income (log) |  |  |  |  | - |  | - |  |
| Household Wealth (log) |  |  |  |  | - |  | - |  |
| Education (years) |  |  |  |  | - |  | - |  |
| *Neighborhood Characteristics* | |  |  |  |  |  |  |  |
| SES Advantage |  |  |  |  | - |  | - |  |
| SES Disadvantage |  |  |  |  | - |  | - |  |
| Built Environment |  |  |  |  | - |  | - |  |
| % Same Household |  |  |  |  | - |  | - |  |
| % Over 65 |  |  |  |  | - |  | - |  |
| % Rural |  |  |  |  | - |  | - |  |
|  | | | | | |  |  |  |
|  | | | | | |  |  |  |

Notes. Model 2 includes controls for wave of interview, age, race and ethnicity, nativity, and marital status.
