# Supplementary material for: Is Childhood Socioeconomic Status Independently Associated with Adult BMI after Accounting for Adult and Neighborhood Socioeconomic Status?
Source: PLoS One. 2017 Jan 17;12(1):e0168481. doi: 10.1371/journal.pone.0168481 (PMC5241009; doi:10.1371/journal.pone.0168481)
Supplement: S4 Table — (DOCX) [file pone.0168481.s004.docx]

|  | Model 1 | | Model 2 | | Model 3 | | Model 4 | |
| --- | --- | --- | --- | --- | --- | --- | --- | --- |
| Variance Components |  | p |  | p |  | p |  | p |
| Between Tract | 0.07(0.05) | 0.0523 | 0.06(0.05) | 0.0851 | - |  | - |  |
| Pseudo *R*^2^ and Goodness of Fit |  |  |  |  |  |  |  |  |
| *R*^2^, Between Tract | - |  | 0.14 |  |  |  |  |  |
| -2Log Likelihood | 7352.31 |  | 7095.74 |  | - |  | - |  |
| AIC | 7358.31 |  | 7141.74 |  | - |  | - |  |
| N, Level 2 | 3124 |  | 3077 |  | - |  | - |  |
| N, Level 1 | 5544 |  | 5422 |  | - |  | - |  |

S4 Table. Variance Components and Model Fit Statistics, HRS 2006-2008
